# The association of sleep duration and quality with depressive symptoms in older Chinese women

**DOI:** 10.1371/journal.pone.0262331

**Published:** 2022-03-15

**Authors:** Liang Ding, Luyao Zhang, Yufei Cui, Qiang Gong, Jiameng Ma, Yongxiang Wang, Haiyun Sang

**Affiliations:** 1 Department of Physical Education, Southeast University, Nanjing, Jiangsu Province, People’s Republic of China; 2 Institute of Exercise Epidemiology and Department of Physical Education, Huaiyin Institute of Technology, Huaian, Jiangsu Province, People’s Republic of China; 3 Department of Medicine and Science in Sports and Exercise, Tohoku University Graduate School of Medicine, Sendai, Japan; 4 Faculty of Physical Education, Sendai University, Miyagi, Japan; Kasturba Medical College Mangalore, INDIA

## Abstract

Poor sleep quality or short and long sleep duration are associated with many negative health outcomes, such as diabetes, hypertension, and fatigue, which may directly or indirectly correlate with poor mental health. Although, the association between sleep duration and quality, and depressive symptoms has been examined, the results of these studies were inconsistent and evidence specifically on older women is lacking. Therefore, we designed a cross-sectional study to evaluate the association between sleep duration and quality, and depressive symptoms in a relatively large sample of older Chinese women. The data were collected from 1,429 older women aged ≥60 years during bone-health examinations in Shanghai. Information on sleep duration and quality were assessed using a self-reported questionnaire. Depressive symptoms were assessed using the Zung self-rating depression scale (SDS), and depressive symptoms were considered present for SDS scores ≥ 45. Logistic regression models were used to analyze the association between sleep and depressive symptoms. After adjusting for all potential confounding factors, a J-shaped association was found between sleep duration and depressive symptoms. When a sleep duration of 6–8 hours was set as a reference, the odds ratios and 95% confidential intervals of short and long sleep duration were 1.31 (0.99, 1.73) and 2.10 (1.40, 3.16), respectively. Moreover, sleep quality was inversely associated with the prevalence of depressive symptoms (*p* for trend = 0.040). When the SDS cut-off score defining depressive symptoms was changed to 40 and 50, these associations were somewhat weakened, but the trend did not change. This study replicated and extended prior research findings that sleep duration and quality may influence mental health in older women.

## Introduction

Sleep plays an important role in maintaining the functions of the human body, such as the maintenance of consciousness, cognitive functions, maintenance of biological rhythms, repair and defense functions, and stress relief [1]. Poor sleep quality is associated with poor health outcomes such as a higher prevalence of hypertension [2] and increased risk of mortality in older populations [3]. On the other hand, many studies have also shown a U-shaped association between sleep duration and health, with some asserting that a sleep duration of 7–8 hours is associated with lower rates of mortality [4] and diabetes [5]. Additionally, some have shown that the risks for stroke, asthma attacks, and cognitive impairment were lower in persons sleeping 6–8 hours at night [6–8]. Sleep durations of less than 6 hours were also associated with an increased prevalence of hypertension [9]. Based on these previous findings, 6–8 hours of sleep could be considered beneficial to human health. However, in older adults, complaints about sleep-related issue are common, of which many are physiological, such as an increased time to fall asleep and decreased total sleep time, and are associated with the normal aging process. Moreover, the incidence of primary sleep disorders is increased in the older adults. Therefore, sleep conditions in older adults differ from those in the general population.

Meanwhile, depressive symptoms are a common mental disorder worldwide [10], and affect peoples’ health, and are associated with cardiovascular diseases [11], diabetes [12], and even mortality among older adults [13]. Thus, preventing or improving depressive symptoms is important for human health. As sleep status is related with many diseases, we speculated that it may also directly or indirectly influence mental health. Some studies have reported a relationship between sleep duration and quality, and depressive symptoms. However, most of these studies focused on a specific population, such as adolescents and young adults. To our knowledge, very few studies have investigated the association of sleep duration and quality with depressive symptoms in older people, especially in elderly Chinese.

The Chinese population accounts for approximately 20% of the total world population. In China, one-third of the elderly aged 75 and older are living with depressive symptoms [14]. In addition, in 2017 there were 240.9 million people aged ≥ 60 years in China [15], and this number is increasing. As sleep states vary with age, it is crucial to examine the association between sleep and depressive symptoms in older populations. Furthermore, the evidence suggests that women have a higher risk of depressive symptoms than men [16, 17]. As it is especially necessary to examine this association with depressive symptoms in women, we designed a cross-sectional study to examine the association of sleep duration and quality with depressive symptoms among older Chinese women. Based on previous studies, we hypothesized that, in addition to the younger population, poor sleep quality or short and long sleep durations would likely be associated with depressive symptoms in older women.

## Materials and methods

### Participants

The present study is a cross-sectional study of a population of older Chinese women. The data were collected from a bone-health examination of women aged 60 years and older at the health-management center in the Jiuhua Area, Shanghai, between April 2019 and May 2019. Participants participated in the health examination voluntarily. An additional questionnaire survey was performed after the examination with one-to-one support. Written consent was obtained from the participants before the survey. The study was approved by the Ethics Committee of the Huaiyin Institute of Technology. We invited all participants who had undergone the bone-health examination, of whom 1,510 agreed to participate. We excluded participants who were taking antidepressants, were receiving psychological therapy (*n* = 42), or who had missing data in their questionnaire (*n* = 39). Therefore, the final population comprised 1,429 elderly women.

### Assessment of sleep

Sleep duration and quality were assessed using a self-reported questionnaire that included sleep-related questions. Participants were asked to rate their difficulties in initiating and maintaining sleep on a five-point scale: 1, *< 1 day per month*; 2, *1–3 days per month*; 3, *4–7 days per month*; 4, *8–15 days per month*; and 5, *≥ 16 days per month*. Subsequently, 1 and 2 were defined as “good sleep,” 3 was defined as “common sleep quality,” and 4 and 5 were defined as “poor sleep quality” [18]. Sleep duration was assessed by the question: “How many hours did you usually sleep at night in the past month?” The response options were: *< 6 hours*, *6–8 hours*, and *> 8 hours*.

### Assessment of depressive symptoms

We assessed depressive symptoms using the Chinese version of the Zung self-rating depression scale (SDS), a self-administered questionnaire designed to screen for the severity of the depressive symptoms that consists of 20 items scored from 1 to 4 points depending on the frequency of the listed problems [19]. The overall score is the sum of the scales corresponding to the 20 items, and ranges from 20 to 80. We used the cut-off value of 45 to define depressive symptoms [20], and the cut-off values of 40 and 50 were used for sensitivity analysis [21]. These values are widely used in epidemiological studies to diagnose depressive symptoms [20, 21]. Greater values indicate increased severity of depressive symptoms. The reliability and validity of the SDS in Chinese populations have been demonstrated by a previous study [22].

### Confounding factors

Body mass index (BMI) was calculated as weight (kg) divided by height (m) squared. Body weight and height were measured before bone examinations and expressed in kilograms and centimeters, respectively. Blood pressure was measured on the upper left arm with an automatic device (KENTARO HBP-9021J, Japan). If the value of the first measurement was abnormal, a second measurement was carried out. Hypertension was defined as systolic blood pressure ≥ 140 mmHg or diastolic blood pressure ≥ 90 mmHg, or the use of an anti-hypertensive drug [23]. Information on age, former occupation, smoking and drinking status, household income, living condition, and educational level were obtained from the questionnaire survey. Former occupation was divided into white-collar and blue-collar occupations. Tobacco smoking was divided into smoker, former smoker, and non-smoker. Alcohol drinking was divided into drinking every day, drinking occasionally, and non-drinker. Household income was divided into three categories: low income ≤ 50 000 Yuan, middle income 50 001–70 000 Yuan, and high income > 70 000. Educational level was divided into < high school and ≥ high school. Physical activity was evaluated by frequency of physical exercise, where more than six days per week was defined as “high physical activity,” one day to five days per week was defined as “middle physical activity,” and “never exercise” was defined as “low physical activity.”

### Statistical analyses

The differences in variables between the depressive symptom categories were examined using analysis of variance (ANOVA) for continuous variables or the chi-square test for categorical variables. A logistic regression analysis was performed to estimate crude odds ratios (ORs) and confidence intervals (CIs) for the association between sleep and depressive symptoms. A multiple logistic regression analysis was performed to adjust for confounding factors. Depressive symptoms were used as the dependent variables, and sleep duration and quality were used as the independent variables. The adjusted model used age, BMI, occupation, smoking and alcohol drinking status, household income, living condition, educational level, hypertension, diabetes, and physical activity as confounding factors. A *p*-value of less than 0.05 was considered statistically significant for all analyses. All analyses were conducted using SPSS version 24.0 (SPSS, Inc., Chicago, IL).

## Results

The detailed characteristics of all participants with or without depressive symptoms are shown in Table 1. After excluding for participants who did not meet the criteria, a total of 1,429 participants were included in the final analysis (mean age 69.2±7.09 years). The rate of depressive symptoms was 24.6% for SDS ≥ 45. The participants with depressive symptoms were more likely to be older (*p* < 0.001), and have a higher BMI (*p* = 0.002). The proportion of blue-collar workers, low household income, low educational level, and diabetes were higher in the depression category (*p* = 0.001, < 0.001, = 0.017, and = 0.008, respectively). Participants who were non-smokers or who had a higher household income were more likely to have less depressive symptoms (*p* < 0.001, and < 0.001, respectively).

**Table 1:** Participant characteristics according to depressive symptoms.

	Non depressive symptoms	Depressive symptoms	p value^a^
	n = 1077	n = 352	
Age (years)	68.6 (68.1, 69.0)	71.1 (70.4, 71.8)	< 0.001
BMI (kg/m^2^)	24.1 (23.9, 24.3)	24.6 (24.3, 25.0)	0.002
Former occupation (n; %)			
White collar	208 (19.3)	28 (8.0)	< 0.001
Blue collar	869 (80.7)	324 (92.0)	
Smoking (n; %)			
Smoker and former smoker	17 (1.6)	14 (4.0)	0.011
Non-smoker	1060 (98.4)	338 (96.0)	
Alcohol drinking (n; %)			
≤ 1 time/week	65 (6.0)	22 (6.2)	0.898
Non-drinker	1012 (94.0)	330 (93.8)	
Household Income (n; %)			
Low	359 (33.3)	157 (44.6)	< 0.001
Middle	349 (32.4)	88 (25.0)	
High	369 (34.3)	107 (30.4)	
Living along (n; %)			
Yes	86 (8.0)	30 (8.5)	0.737
No	991 (92.0)	322 (91.5)	
Educational level (n; %)			
≥ High school	217 (20.1)	93 (26.4)	0.017
< High school	860 (79.9)	259 (73.6)	
Hypertension (n; %)			
Yes	63.4 (59.7)	195 (55.4)	0.170
No	434 (40.3)	157 (44.6)	
Diabetes (n; %)			
Yes	176 (16.3)	80 (22.7)	0.008
No	901 (83.7)	272 (77.3)	
Physical activity (n; %)			
Low	397 (36.9)	199 (56.5)	< 0.001
Middle	141 (13.1)	65 (18.5)	
High	539 (50.0)	88 (25.0)	

^a^ Obtained by using ANOVA for continuous variables and x^2^ test for variables of proportion.

We assessed the association between sleep duration and depressive symptoms in logistic regression models, from which the odds ratios and 95% CIs of the crude and adjusted models are shown in Table 2. No linear association was found between sleep duration and depressive symptoms. However, compared with a sleep duration of 6–8 hours, the odds ratio and 95% CIs of < 6 hours and > 8 hours sleep duration were 1.37 (1.06, 1.79) and 1.94 (1.33, 2.83), respectively (*p* < 0.05 for both) in the crude model. In addition, this J-shaped association did not change in Model 1. In the final adjusted model (Model 2), greater than 8 hours of sleep was significantly associated with a higher prevalence of depressive symptoms.

**Table 2:** Adjusted associations between sleep duration and depressive symptoms among 1,429 older women.

	Sleep duration (hours)			p for trend^a^
	< 6	6–8	> 8	
n.	484	794	151	
Depressive symptoms, n	131	169	52	
Crude	1.37 (1.06, 1.79)^b^^,^ ^c^	1	1.94 (1.33, 2.83)^c^	0.766
Model 1^d^	1.32 (1.01, 1.72)^c^	1	2.11 (1.44, 3.11)^c^	0.413
Model 2^e^	1.31 (0.99, 1.73)	1	2.10 (1.40, 3.16)^c^	0.458

^a^ Obtain using Multiple logistic regression analysis.

^b^ Results are expressed as odds and 95% CIs (all such variables).

^c^ Significantly different to the reference category (p < 0.05).

^d^ Adjusted for age, BMI.

^e^ Adjusted for age, BMI, educational level, former occupation, household income, living condition, smoking and drinking habits, hypertension, diabetes, physical activity.

The association between sleep quality and depressive symptoms is shown in Table 3. Compared to good sleep quality, normal and poor categories of sleep quality were significantly associated with a higher prevalence of depressive symptoms, showing a clear inverse linear trend in the crude model (*p* for trend = 0.029). A similar association was observed in Model 1; the adjusted ORs (95% CIs) for depressive symptoms across all categories of sleep quality were 1 for good sleep quality, 1.06 (0.79, 1.44) for normal sleep quality, and 1.44 (0.99, 2.10) for poor sleep quality, (*p* for trend = 0.071). In Model 2, this inverse association was unchanged (*p* for trend = 0.040).

**Table 3:** Adjusted associations between sleep quality and depressive symptoms among 1,429 older women.

	Sleep quality			p for trend^a^
	Good	Normal	Poor	
n.	374	819	236	
Depressive symptoms, n	84	195	73	
Crude	1	1.08 (0.81, 1.44)^b^	1.55 (1.07, 2.23)^c^	0.029
Model 1^d^	1	1.06 (0.79, 1.44)	1.44 (0.99, 2.10)	0.071
Model 2^e^	1	1.31 (0.92, 1.85)	1.68 (1.01, 2.79)^c^	0.040

^a^ Obtain using Multiple logistic regression analysis.

^b^ Results are expressed as odds and 95% CIs (all such variables).

^c^ Significantly different to the first category (p < 0.05).

^d^ Adjusted for age, BMI.

^e^ Adjusted for age, BMI, educational level, former occupation, household income, living condition, smoking and drinking habits, hypertension, diabetes, physical activity, sleep duration.

**Fig 1:**
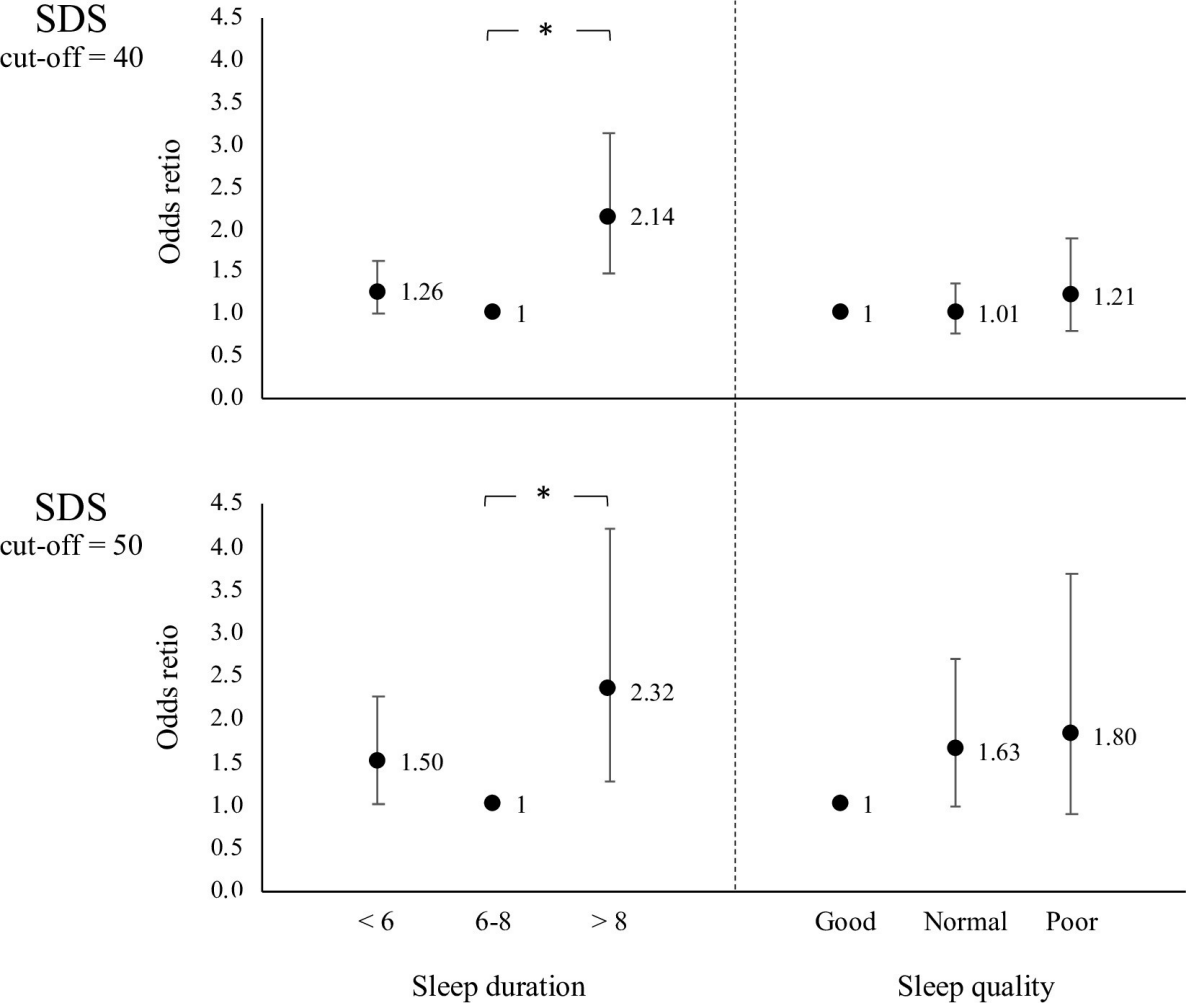
Adjusted association between sleep duration, sleep quality and depressive symptoms in 1,429 older women. Results was adjusted using age, BMI, educational level, former occupation, household income, living condition, smoking and drinking habits, hypertension, diabetes, physical activity. Additionally, adjusted using sleep duration for sleep quality. *: p <0.05.

The results of the sensitivity analysis using SDS cut-off of 40 and 50 points are shown in Fig 1. Sleep durations greater than 8 hours were significantly associated with a higher prevalence of depressive symptoms for both SDS cut-off points, 40 and 50. On the other hand, although it was not significant, there was an inverse association between sleep quality and the prevalence of depressive symptoms at both SDS cut-off points, 40 and 50.

## Discussion

In the present study, we investigated the association of sleep duration and quality with depressive symptoms among older Chinese women. The results showed that sleep durations of less than or equal to 8 hours and good sleep quality were associated with a lower prevalence of depressive symptoms. Our study expands previous findings on the association of sleep and depressive symptoms and suggests that sleep duration and quality may influence mental health in older women.

To our knowledge, this is the first study to investigate the association between sleep duration and depressive symptoms in older Chinese women. Our present findings are consistent with studies verifying that short or long sleep durations are associated with a higher prevalence of depressive symptoms among older populations. An American cohort study found that short sleep duration was associated with a higher risk of depressive symptoms in 1,110 older adults aged 65 years and older [24]. Another study showed that long sleep duration was associated with a higher risk of depressive symptoms in 2,510 elderly American men aged 65 years and older [25]. A Japanese longitudinal study also showed that long sleep duration was associated with a higher risk of depressive symptoms in 4,997 elderly people aged 65 years and older [26]. In addition, a study in the Netherlands conducted with 5,019 persons aged 58–100 years found that both short- and long-duration sleepers were more likely to have a depressive disorder [27]. However, sex-stratified analyses were not performed in these studies. Thus, these results only hold for the older population in general and for older men. Considering that older women who have experienced menostasis have different sleep patterns and a different mental status than men, this association merits investigation in elderly women. In fact, one American study indicated that both short (< 5 hours) and long sleep (> 8 hours) duration were associated with increased odds of depressive symptoms in 952 women aged 70 years and older [28]. However, there are some differences, such as age, nationality, and assessment of depressive symptoms, between that study and ours. Our study thus helps make up for the deficiency in studies on women.

Studies of adolescents and adults have suggested that the risk of depressive symptoms increases with poor sleep quality. A cross-sectional study found that sleep disturbance was a predictor of depressive symptoms in 1,500 female Chinese nurses [29]. Additionally, in another Chinese study of 17,946 adolescents (14–18 years old), sleep difficulties were verified to be positively correlated with depressive and anxiety symptoms in both cross-sectional and longitudinal samples [30]. A few studies have examined this association in the elderly population. They found that sleep disturbance at baseline increased odds of depressive symptoms at follow-up a few years later [28, 31]. On the other hand, one study found that good self-reported sleep quality was related to low scores on the PHQ-9 (nine-item Patient Health Questionnaire) scores (β = 0.59, *p* < 0.001), in an older Chinese population (age ≥ 60 years) [32]. Although the evaluation of sleep duration, quality, and depressive symptoms in our study differs from these previous studies, our study is consistent with them, and further strengthens the evidence of an association of sleep duration and quality with depressive symptoms in older Chinese women.

There are several potential explanations for the association between sleep status and depressive symptoms. First, a major explanation is inflammation, a key factor strongly associated with depression [33]. Studies have reported both short and long sleep durations and poor sleep quality to be associated with increased inflammatory cytokines such as CRP and IL6 [34–36]. Second, good sleep quality could help increase levels of melatonin [37], a pleiotropic regulator molecule that has been reported to alleviate depressive symptoms [38]. Third, in older adults, poor sleep quality potentially induces more negative cognitions, emotions and activities, which eventually results in a greater depressive symptom [39]. Fourth, a previous study suggested that poor sleep contributes to a decline in physical functioning among older women [40], and recent evidence suggests that sleep problems may increase the risk of chronic pain [41]. Furthermore, these two factors may be associated with a high risk of depressive symptoms.

The strength of this study is that we used three cut-off points in the SDS score (from mild to severe) to examine the association with sleep. On the other hand, there are also several limitations to our study that must be considered. First, due to the nature of cross-sectional studies, it is difficult to draw conclusions about causality from our results. Second, participants in this study were drawn from just one bone-health examination program in one area of Shanghai, and individuals taking antidepressants or receiving psychological therapy were excluded. Thus, as a selection bias existed, the present study sample may not be representative of the general population. Further research is required to ascertain whether these associations are replicated in other populations. Third, as data on confounding factors were limited, we cannot exclude the possibility that depressive symptoms are affected by other factors correlated with sleep. Fourth, sleep duration was categorized in the questionnaire as < 6, 6–8, and > 8 hours. Thus, we cannot exclude the possibility that the results that would change were optimal sleep duration to be set at either 6–7 or 7–8 hours. In addition, standardized scales to assess sleep quality, such as the Pittsburgh Sleep Quality Index, could not be used in this study due to limited questionnaire space and survey time. Fifth, we collected data on sleep duration and quality using a self-reported questionnaire. Real sleep conditions were not observed, which introduces the possibility of recall bias.

In conclusion, the present study shows that sleep durations less than or equal to 8 hours and good sleep quality may be associated with a lower prevalence of depressive symptoms among older Chinese women. Considering that sleep duration less than 6 hours is not recommended, maintaining a 6–8-hour sleep duration may be optimal to mental health. Our findings further strengthen the evidence on the association between sleep and mental health and provide important evidence for woman’s mental health and implications for the field of preventive medicine and health education. Prospective studies or randomized trials are required to confirm these findings and clarify their causality.
